# Properties and Microstructural Characteristics of Manganese Tailing Sand Concrete

**DOI:** 10.3390/ma15165583

**Published:** 2022-08-15

**Authors:** Min Bai, Guangcheng Long, Fan Wang

**Affiliations:** School of Civil Engineering, Central South University, Changsha 410075, China

**Keywords:** manganese tailing sand, manganese tailing sand concrete, mechanical properties, air void characteristics, microstructure, resource reuse

## Abstract

In this work, manganese tailing sand concrete (MTSC) was prepared using manganese tailing sand (MTS) in replacement of river sand (RS) to alleviate the shortage of RS resources and achieve clean treatment and high-value resource utilization of manganese tailing stone. The effects of MTS content on the slump, mechanical strength, air void characteristics, hydration products and micromorphology of MTSC were studied experimentally. The leaching risk of harmful substances in MTSC was also explored by testing the concentration of Mn^2+^. The results show that the utilization of MTS reduces the slump of MTSC to a certain extent. When the MTS content is lower than 40%, the gypsum introduced by MTS and C_3_A in cement undergoes a hydration reaction to form ettringite, which decreases the number of pores with a diameter less than 0.1 mm and promotes strength development in MTSC. Additionally, when the MTS content exceeds 40%, the large amount of gypsum reacts to form more ettringite. The expansive stress generated by the ettringite severely damages the pore structure, which is not conducive to the mechanical properties of MTSC. In addition, the leaching of hazardous substances in MTSC is insignificant, and the incorporation of cement can effectively reduce the risk of leaching hazardous substances in MTSC. In summary, it is completely feasible to use MTS to replace RS for concrete preparation when the substitution rate of MTS is less than 40%, with no risk of environmental pollution. The results and adaptation in the concrete industry can reduce the carbon footprint, which is in line with the current trend in civil and materials engineering.

## 1. Introduction

In recent years, as the scale of China’s infrastructure construction continues to expand, concrete has become a widely used construction material [1]. In 2020, China produced 2.5 billion m^3^ of concrete, which consumed nearly 1.65 billion tons of sand and gravel, including a large number of natural river sand (RS) [2]. However, since 2018, multiple factors such as China’s restriction of RS mining, a crackdown on illegal sand mining and enhanced environmental protection supervision have led to an oversupply and a continuous price increase for RS. In addition, the over-exploitation of RS has caused frequent geological disasters such as riverbank collapse [3] and has a serious impact on the ecological environment [4]. Therefore, finding suitable materials to replace RS for concrete preparation is beneficial to alleviate the tension of RS resources and protect the ecological environment.

In response to these problems, many studies have been conducted by relevant researchers [5,6,7]. Blessen Skariah Thomas et al. [8] prepared copper tailing concrete using copper tailing as a partial replacement for natural sand. It was found that copper tailing can be used to partially replace natural fine aggregate at the water–cement ratios of 0.4, 0.45 and 0.50 until the replacement rate reaches 60%. Since copper tailings concrete (up to 60% replacement) exhibits good strength and durability characteristics, it can be recommended for all construction activities. Ramesh Chandra Gupta et al. [9] conducted an experimental study on the strength, permeability, abrasion, carbonation and shrinkage properties of concrete based on the inclusion of different percentages of copper tailings as a partial replacement for natural fine aggregates. In the range of 0–80%, concrete prepared by replacing natural sand with copper tailings showed no significant degradation in performance. Concrete containing copper tailings can be recommended to replace 70% of natural fine aggregates for all applications, while substitutions above 70% can be recommended for nonstructural applications, pavements, etc. İlker Bekir Topçu et al. [10] investigated the physical and mechanical properties and freeze-thaw durability of concrete produced using waste concrete aggregates (WCAs). It found that C16-quality concrete could be produced using less than 30% C14-quality WCA. It was also observed that the unit weight, workability and durability of concrete produced by WCA decreased in inverse proportion to their endurance for freeze–thaw cycle. Krzysztof Ostrowski et al. [11] used granite waste (GW) as an alternative aggregate for Self-Compacting High-Performance Concrete (SCHPC). The effect of coarse aggregate morphology on the behavior of fresh concrete was investigated, as well as on the deformation and compressive strength of hardened concrete. The GW morphology was also described using microscopic laboratory tests. Laboratory tests on the properties of fresh concrete mixtures were also performed and found that the aggregate morphology has an important influence on the properties of the fresh mixtures of SCHPC and on the mechanical characteristics of the hardened concrete.

Moreover, as the world’s largest producer, consumer and exporter of manganese metal, China accounts for more than 97% of the global production of manganese metal [12,13]. With the large-scale mining of manganese ore, a large amount of solid waste is generated, mainly including electrolytic manganese residue and manganese tailing stone. Electrolytic manganese residue is the filtrate containing more ammonia nitrogen, sulfide and heavy metals (Mn, Cd, Zn, Cr and V, etc.) produced during the electrolysis of manganese metal [14]. Manganese tailing stone is a stone with low manganese content screened out from manganese ore at the early stage of mining. Relevant data show that every 1 ton of manganese metal produced can produce 10–13 tons of electrolytic manganese residue and 21–23 tons of manganese tailing stone [15,16]. Therefore, with the continuous production of manganese metal, more and more electrolytic manganese residue and manganese tailing stone are produced. At present, many scholars have conducted much research on the harmless treatment and resource utilization of electrolytic manganese residue and achieved certain results [17,18]. However, the research on manganese tailing stone mainly focuses on the recovery of valuable resources [19,20], and there are few studies on the harmless treatment and resource utilization of manganese tailing stone. However, as the grade of manganese ore decreases, the emission of manganese tailing stone increases dramatically. Currently, the main disposal method of manganese tailing stone is landfill, which occupies a large number of land resources. Moreover [21], with the change in environment and time, there is a certain risk of leaching of heavy metals in manganese tailing stone, which can inevitably pollute the environment and endanger human health [22,23]. In addition, the accumulation of manganese tailing stones in landfills also cause waste of resources to a certain extent. Therefore, it is urgent to strengthen the recovery and comprehensive utilization of manganese tailing stones and promote the sustainable development of manganese resources.

In summary, RS, as one of the main raw materials for concrete, has led to frequent geological disasters due to large-scale RS mining as concrete usage has increased dramatically. In addition, with the rapid development of the manganese industry in China, the accumulation of a large amount of manganese tailing stone seriously occupies the land and has the risk of environmental pollution. Therefore, it is urgent to explore the alternatives of river sand and the resource utilization of manganese tailing stones. Based on these, this paper first made manganese tailing stone into manganese tailing sand (MTS) and then used MTS to partially or fully replace river (RS) sand to prepare manganese tailing sand concrete (MTSC). With the content of MTS as a variable, the effect on the slump, mechanical properties, pore characteristics and microstructure of MTSC was studied. The influence of MTS on the hydration of concrete and the risk of leaching harmful components were discussed. A suitable replacement rate of MTS without losing the basic properties of concrete was proposed. The results of the study not only provide a new way for the clean and high-value utilization of manganese tailing stone but also offer a new solution for the transitional consumption of RS.

## 2. Experimental Programs

### 2.1. Raw Material

**Figure 1:**
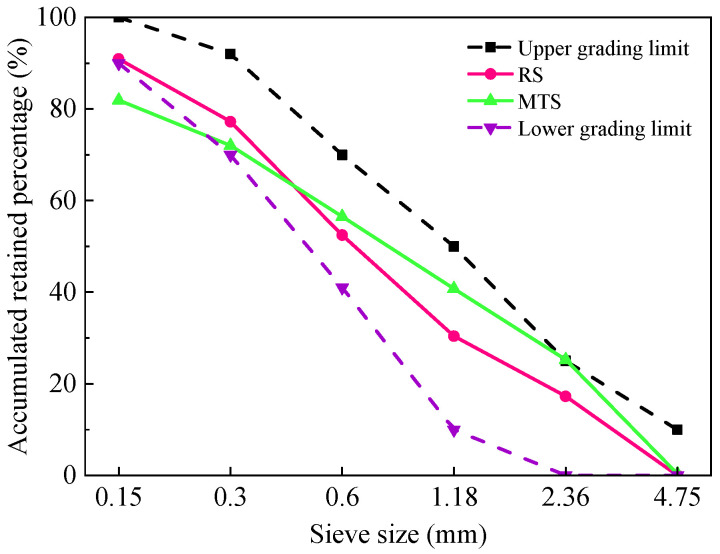
The gradation curves of RS and MTS.

**Figure 2:**
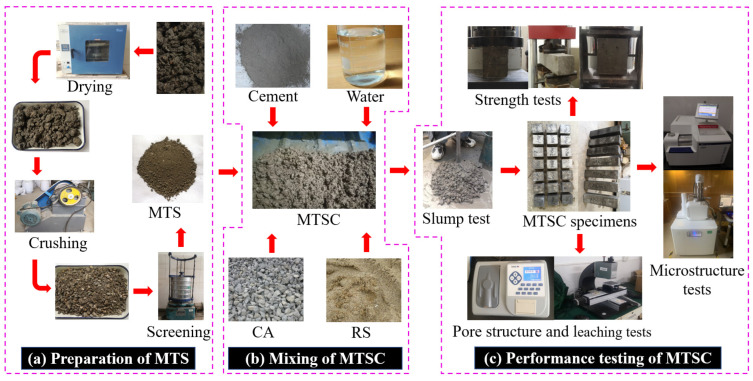
Test process diagram. (**a**) Preparation of MTS, (**b**) Mixing of MTSC, (**c**) Performance testing of MTSC.

**Figure 3:**
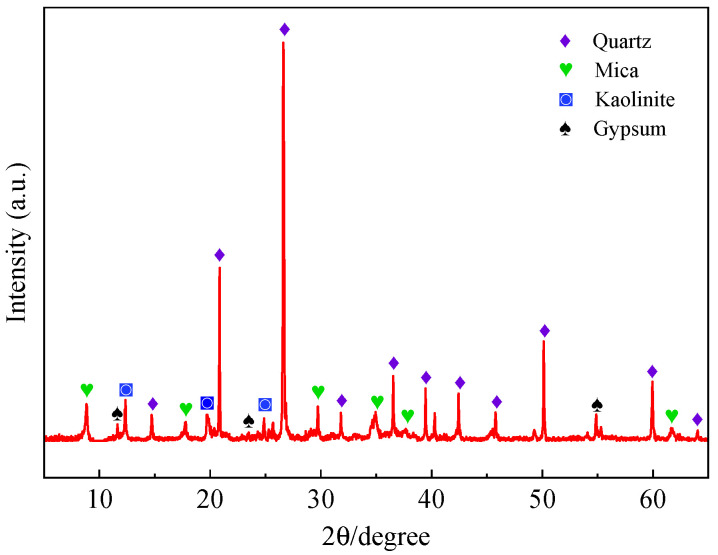
The XRD pattern f MTS.

The cement used in this study was P·O 42.5 ordinary Portland cement. The main properties and chemical compositions of cement are shown in Table 1 and Table 2, respectively. River sand (RS) was purchased from a sand plant in Changsha, Hunan province. The relevant physical properties are shown in Table 3, and the gradation curve is shown in Figure 1. Manganese tailing sand (MTS) was produced by drying, crushing and screening manganese stone. The specific preparation process is shown in Figure 2. The chemical compositions and physical properties are shown in Table 2 and Table 3, and the gradation curve is shown in Figure 1. The XRD pattern of MTS (Figure 3) indicates that the main mineral of MTS is quartz, and it also contains mica, kaolinite and a small amount of gypsum. Coarse aggregate (CA) was continuously graded gravel with a diameter of 5~10 mm. The apparent density of CA is 2750 kg/m^3^, water absorption is 0.68%, and mud content is less than 1%. The concrete mixing water was domestic water.

### 2.2. Mix Proportions

Table 3 shows that the physical properties of MTS are poor, especially the content of stone powder, clay lump content and crushing indexes do not meet the standards of construction sand. It is not feasible to use MTS to completely replace RS to prepare concrete. Therefore, a certain mass fraction (0%, 20%, 30%, 40%, 50%, 60%, 100%) of MTS was selected to replace part of RS to prepare Manganese tailings sand concrete (MTSC). The water–cement ratio and the sand ratio of MTSC were 0.45 and 0.41, respectively. All mix proportions of MTSC are shown in Table 4, where the labels MTSC-0, MTSC-2, MTSC-3, MTSC-4, MTSC-5, MTSC-6 and MTSC-10 represent the MTSC with 0%, 20%, 30%, 40%, 50%, 60% and 100% replacement rates of MTS, respectively.

The preparation process of MTSC is shown in Figure 2. First, the raw materials were weighed according to the mix proportions of MTSC. Then cement and fine aggregate were poured into the mixing pot and stirred for 1 min. Then coarse aggregate was added and stirred for 1 min. Finally, the water was added and continued to stir for 2 min. After mixing, the concrete mixtures were poured out to test the slump. Later the slump test was completed, and the mixtures were immediately loaded into molds. Then, a vibration table was used to make the mixtures dense. Finally, these samples were firstly covered with film for natural curing for 1d, and then the molds were dismantled. These samples were then placed into a standard curing room (curing temperature (20 ± 2) °C, relative humidity above 95%) for curing.

### 2.3. Test Methods

#### 2.3.1. Mechanical Properties

(1) Slump: The slump test of MTSC was carried out according to “the national standard of the People’s Republic of China: Standard test methods for the performance of ordinary concrete mixes (GB/T 50080-2016)” [25].

(2) Strength: The flexural strength (loading rate of 0.2 kN/s), compressive strength (loading rate of 0.5 MPa/s) and splitting tensile strength (loading rate of 0.05 MPa/s) of MTSC were tested by using a microcomputer-controlled constant loading pressure testing machine (TYA-2000E, Wuxi Jianyi Instrument Machinery Limited Company, Wuxi, China)

Additionally, in order to investigate the strength effect of MTS in MTSC, the strength contribution value *K* was introduced to quantify the strength contribution of MTS. Assuming that Equation (1) holds, the strength of MTSC after mixing with *x* content MTS is *σ*, and the strength contribution *K* of MTS can be calculated according to Equation (3) by combining Equations (1) and (2).

(1) $$\sigma_{e}=Κ\sigma_{0}$$

(2) $$\sigma =x\sigma_{e}+(1-x)\sigma_{0}$$

(3) $$K=(\sigma /\sigma_{0}+x-1)/x$$

where *K* is strength contribution value; *x* is the dosage of MTS; *σ*_0_ is the strength of concrete without MTS, MPa; *σ*_e_ is the strength contribution of unit MTS, MPa; *σ* is the strength of concrete mixed with *x* content MTS, MPa;

(3) Pore structure: The MTSC sample with an age of 28 d was cut into slices, and then the uneven surfaces of these slices were polished using a grinding machine. Then, the surfaces of the specimen were coated with black ink, and the mixture of ZnO and petroleum jelly was applied again after the surfaces of the specimen were dried. The excess coating on the surfaces was wiped off when the holes on the surface of the specimen were completely filled. The hardened concrete, porous structure analyzer (CABR-457, Zhongxi Yuanda Technology Co., Ltd., Beijing, China) was used to analyze the characteristics of the pore structure of MTSC;

(4) Leaching of harmful substances: The leaching test of MTSC was carried out by the horizontal oscillation extraction process. After crushing and grinding the samples to a particle size of less than 0.075 mm, then 5 g of the sample was placed into a beaker and stirred for 2 min in a magnetic stirrer with a solid–liquid ratio of 1:10. The sample was then placed on a constant temperature shaker and shaken at a frequency of 160 min^−1^ for 10 h and left for 14 h. Finally, the supernatant was taken to test the content of Mn^2+^ by using the water quality tester (5B-3BW, Shandong Hongde Industrial Co., Ltd., Jining, China).

#### 2.3.2. Microstructure

MTSC specimens were crushed after curing to 28 d. Samples with a particle size less than 5 mm were taken, and hydration was prevented by using isopropyl alcohol. MTSC flake samples were taken out, and their hydration products were observed using scanning electron microscopy (SEM). The remaining samples were ground in a mortar and passed through a 0.075 mm sieve. The samples were analyzed by X-ray diffraction (XRD, CuKα radiation, 40 kV, 40 mA, scan speed 2°/min, scan range 5~70°) and thermogravimetry (TG/DTG, 30~1000 °C, 10 °C/min and N_2_ environment) for the composition of the physical phases and mineral phase alterations.

## 3. Results and Discussion

### 3.1. The Fluidity of Fresh MTSC

**Figure 4:**
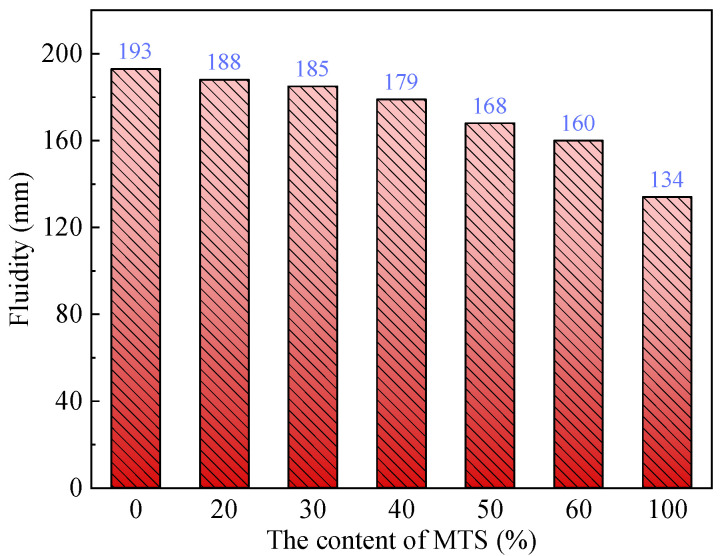
Effect of MTS content on fluidity of MTSC.

The effect of MTS content on MTSC fluidity is shown in Figure 4. It can be seen that with the increase in MTS content, the fluidity of MTSC continues to decline. Without MTS, the fluidity of concrete is 193 mm; the content of MTS is 100%, the fluidity of concrete decreases to 134 mm and the decrease rate is 30.6%. The content of MTS has a great influence on the fluidity of MTSC. The main reason is that, on the one hand, MTS has more fine powder content and requires more water; on the other hand, MTS may produce tiny cracks when it is crushed, which leads to a greater water absorption rate of MTS than RS; thus, the fluidity of MTSC decreases with the increase in MTS content. It is worth noting that when the content of MTS is less than 40%, the fluidity of MTSC decreases by less than 7.4%, and the fluidity of MTSC basically meets the construction requirements.

### 3.2. The Strength of MTSC

#### 3.2.1. Flexural Strength

**Figure 5:**
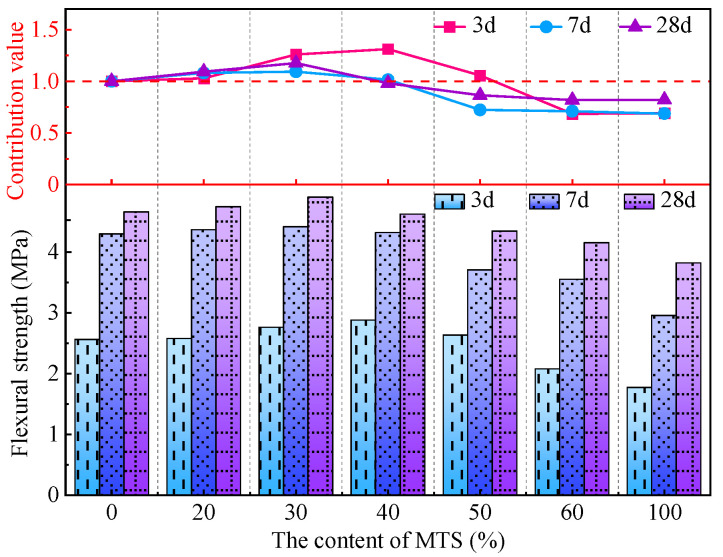
Effect of MTS content on flexural strength of MTSC and strength contribution value.

Figure 5 shows the effect of MTS content on the flexural strength of MTSC and its strong contribution value. The flexural strength of MTSC at different ages increases first and then decreases with the increase in MTS content. The flexural strength of MTSC-0 at 3 d and 28 d is 2.6 MPa and 4.7 MPa, respectively. Compared with MTSC-0, when the MTS content increases from 20% to 100%, the growth rates of flexural strength of MTSC at 3 d and 28 d is 0.6%, 7.8%, 12.5%, 2.8%, −18.9% and −31.0% and 1.9%, 5.3%, −0.8%, −6.8%, −10.9% and −18.0%, respectively. It can be seen that the flexural strength of MTSC is improved when the content of MTS is not more than 40%. However, when the content of MTS is more than 40%, it is not beneficial to the development of concrete’s flexural strength. Especially when the content of MTS is 100%, the flexural strength of MTSC decreases significantly. With the increase in MTS content, on the one hand, the fine powder content of sand used in MTSC also increased, and the filling effect of micronized powder leads to a denser internal structure of MTSC, which helps the strength development of MTSC, but the excessive fine powder can lead to the reduction in the sand in MTSC, and the skeleton of concrete is not solid, thus the strength deterioration. On the other hand, because the crushing value of MTS is larger than RS, with the increase in MTS, the crushing value of sand used in MTSC increases gradually, crushing index of too large is not conducive to the development of concrete strength.

Figure 5 also shows that the dosage of MTS is less than 40%, and the strength contribution value of each age is greater than 1.0. Specifically, at the age of 3 d, the strength contribution value of MTS with 40% content reaches a maximum of 1.31. Moreover, the strength contribution value is above 1.0, with the content of MTS less than 50%. However, when the content is 100%, the strength contribution value drops to 0.69. At the age of 7 d, the strength contribution value of 30% MTS reaches its maximum of 1.09. However, at the age of 28 d, the strength contribution value of MTS with 100% content is the largest.

#### 3.2.2. Compressive Strength

**Figure 6:**
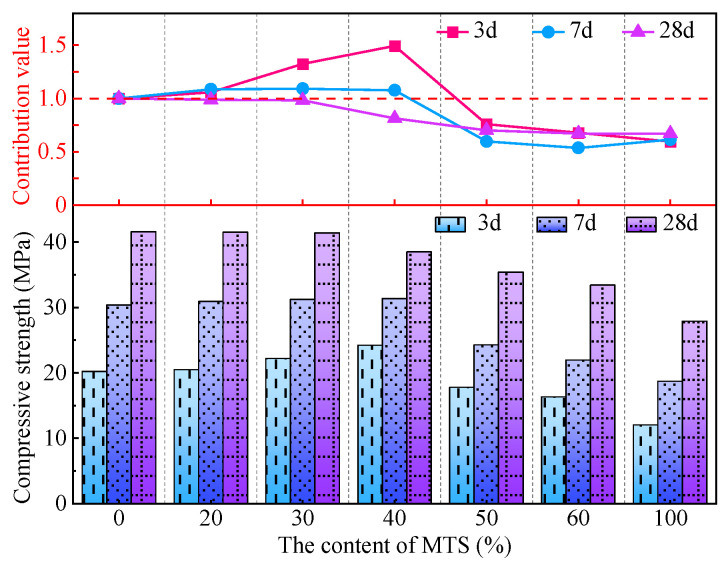
Effect of MTS content on compressive strength of MTSC and strength contribution value.

The influence of MTS content on the compressive strength of MTSC and its strong contribution value is shown in Figure 6. With the increase in MTS content, the compressive strength of MTSC at different ages presents inconsistent regularity. The compressive strength of MTSC at 3 d and 7 d increases first and then decreases with the increase in MTS content. However, the compressive strength at 28d decreases gradually with the increase in MTS content. Specifically, when the age is 3 d and 7 d, the compressive strength of MTSC with 40% MTS content reaches its maximum value, which is 24.2 MPa and 31.3 MPa, respectively. When the age is 28 d, the compressive strength of concrete without MTS is the highest, which is 41.6 MPa. The results indicate that MTS with certain content (content ≤ 40%) can improve the early compressive strength of concrete, but there is no obvious advantage in the later compressive strength development. Too much (content > 40%) of MTS has no contribution to the compressive strength of concrete. The compressive strength of MTSC with an MTS content of 100% is much lower than that of concrete without or with a small amount of MTS. The main reason is that part of gypsum is contained in MTS. Gypsum can react with C_3_A in cement to form ettringite and fill micropores, making the concrete structure dense and conducive to the development of early compressive strength. However, due to excessive gypsum, a lot of ettringite in MTSC forms expansion stress, leading to the decline of compressive strength. Another reason is that the increase in MTS content leads to an increase in fine powder content and a higher crushing index of the sand. The last reason is that the increased particle crushing index of MTS forms a weak microaggregate effect compared to RS. It is worth noting that the 28 d compressive strength of MTSC with 20% and 30% MTS is 41.5 MPa and 41.4 MPa, and the decrease rate is only 0.24% and 0.48%. In addition, the growth rate of the compressive strength of MTSC is obviously faster in the early stages than in the middle and late stages, which is consistent with the growth law of the compressive strength of ordinary concrete [26,27]. This indicates that RS can be replaced by MTS to prepare concrete, but the replacement rate should be less than 40%. Otherwise, it can lead to a substantial decline in the strength of concrete.

Figure 6 also shows that both age and dosage cause changes in the strength contribution value of MTS to concrete. When the content of MTS is less than 40%, the strength contribution value of 3 d and 7 d is greater than that of 28 d, especially the strength contribution value of 3d is as high as 1.49. When the MTS content is 40~100%, the strength contribution value is 3 d > 28 d > 7 d. It is worth noting that when the MTS content is 100%, the strength contribution value of 28 d is the largest, which is 0.67.

#### 3.2.3. Splitting Tensile Strength

**Figure 7:**
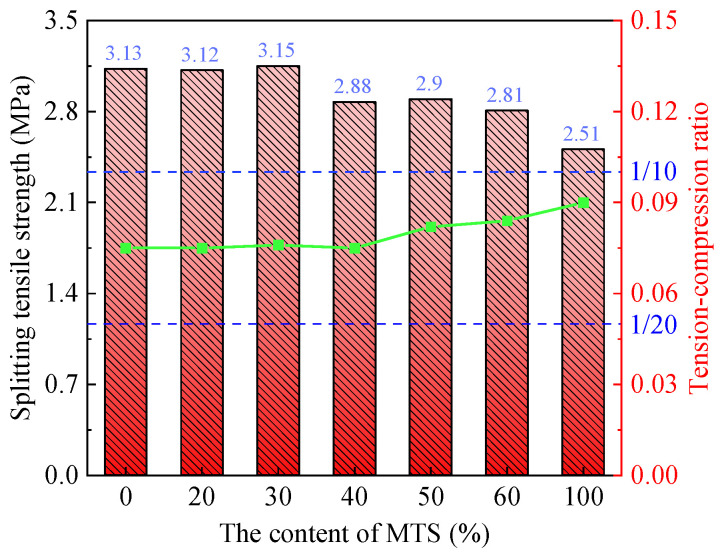
Effect of MTS content on splitting tensile strength and tension–compressive ration of MTSC.

Figure 7 shows the influence of MTS content on the 28d splitting tensile strength and the tension–compression ratio of MTSC. As can be seen from Figure 7, when the content of MTS is less than 30%, the splitting tensile strength of MTSC remains unchanged basically. However, when the content of MTS is more than 30%, the splitting tensile strength of MTSC decreases gradually with the increase in MTS. Moreover, the ratio of splitting tensile strength to compressive strength of MTSC remains between 1/10 and 1/20, which is consistent with the law of ordinary concrete [28,29]. In conclusion, the addition of an appropriate amount of MTS cannot degrade the splitting tensile strength of concrete and may even slightly improve it. Additionally, the addition of excessive MTS leads to a significant decrease in the splitting tensile strength of concrete.

### 3.3. The Pore Structure Characteristics of MTSC

The air void distribution and air content of the hardened MTSC are measured by the air void structure analyzer CABR-457, and the pore areas are calculated by the CABR-457 based on the gray value difference.

**Figure 8:**
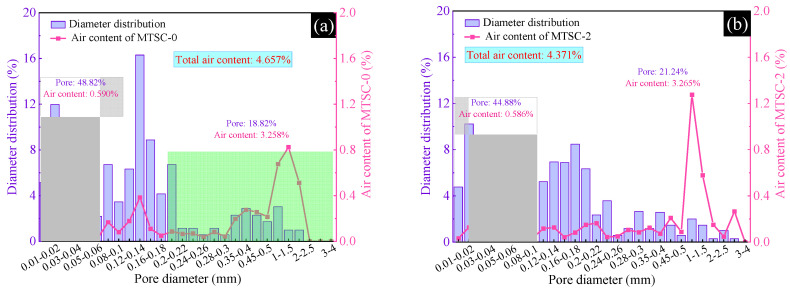

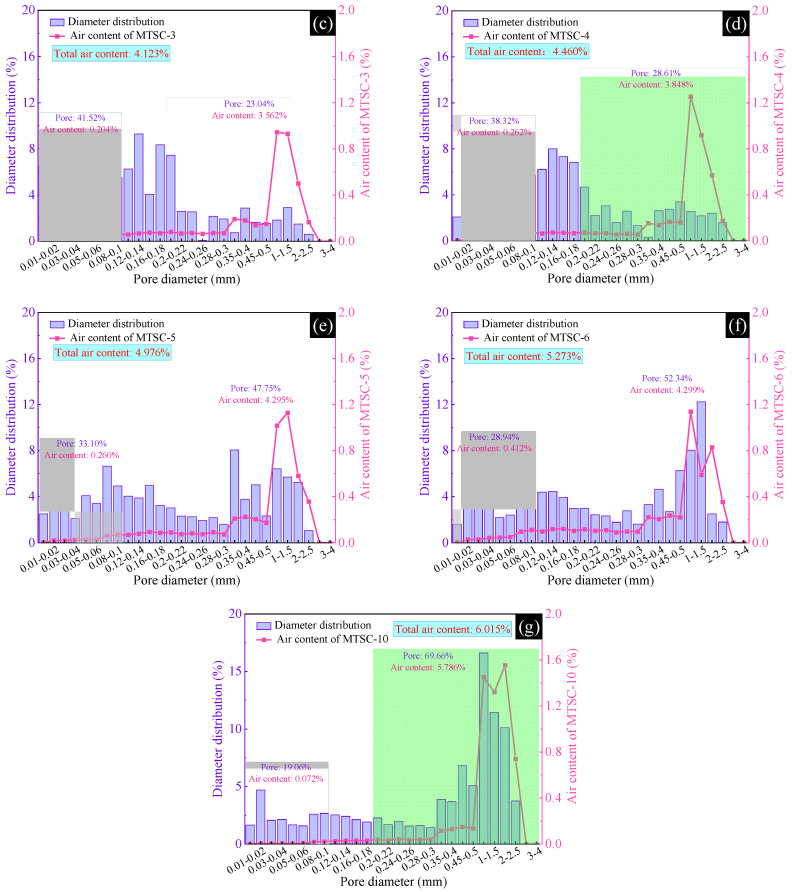
Pore diameter distribution and air content of MTSC. (**a**) MTSC-0, (**b**) MTSC-2, (**c**) MTSC-3, (**d**) MTSC-4, (**e**) MTSC-5, (**f**) MTSC-6, (**g**) MTSC-10.

The air void diameter distribution and air content of the hardened MTSC are shown in Figure 8. Existing studies show that the reduction in pores with a diameter less than 0.1 mm and the increase in pores with a diameter more than 0.2 mm would reduce the strength of the concrete [30,31]. As shown in Figure 8a–g, with the increase in MTS dosing, the pores with a diameter less than 0.1 mm in MTSC gradually decrease, while the pores with a diameter more than 0.2 mm gradually increase. Specifically, with the increase in MTS content from 0% to 100%, the number of pores with a diameter less than 0.1 mm in hardened MTSC decreases from 48.82% to 19.06%, and the reduction rate is 61.0%. The quantity of pores with a diameter greater than 0.2 mm increases from 18.82% to 69.66%, and the rate of increase is 270.1%. The main reason is that, on the one hand, the content of fine powder (particle size < 0.15 mm) in MTS is high, and the fine powder plays a filling role in MTSC, which makes its structure more compact and reduces the small pores (diameter < 0.1 mm). However, the increase in MTS content can lead to the reduction in the content of sand with a particle size of 0.15~0.3 mm, so the large pores (diameter > 0.2 mm) in MTSC increase. On the other hand, MTS contains a small amount of gypsum, which can react with cement to form ettringite, thus effectively filling the pores of the concrete. However, with excess MTS, the large amount of ettringite produced by the reaction can generate swelling stresses, which can destroy some of the small pores and interconnect to form large pores, resulting in more pores with a diameter of more than 0.2 mm in MTSC. It is noteworthy that compared with MTSC-0, the pores with a diameter less than 0.1 mm in the hardened MTSC are reduced by 7.30%, and the reduction rate is 15.0% when the amount of MTS does not exceed 40%. Additionally, the pores with a diameter larger than 0.2 mm increase by 9.79%, with an increase rate of 52.0%. Moreover, the distribution of pores in MTSC is more uniform with the incorporation of a moderate amount of MTS.

Figure 8 also shows that the air content of hardened MTSC is as follows: MTSC-3 < MTSC-2 < MTSC-0 < MTSC-4 < MTSC-5 < MTSC-6 < MTSC-10. MTSC-3 has the lowest air content, while MTSC-10 has the highest air content. Compared with MTSC-0, the air content of MESC-3 is 4.123%, which is reduced by 0.534%. As the gypsum in MTS reacts with the cement, excess free water is consumed. Thus, MTSC-3 forms fewer capillary pores during the hardening process, and it has the least air content. In contrast, the air content of MTSC-10 is 6.015%. This may be due to the stress generated by excess ettringite, which causes the pores in MTSC-10 to be interconnected. Therefore, a large number of capillary pores are formed inside the concrete, and the air content of MTSC-10 becomes larger. In conclusion, the air content of hardened MTSC again confirms the strength variation law of MTSC.

### 3.4. The Hydration Phase of MTSC

#### 3.4.1. TG/DTG and XRD

**Figure 9:**
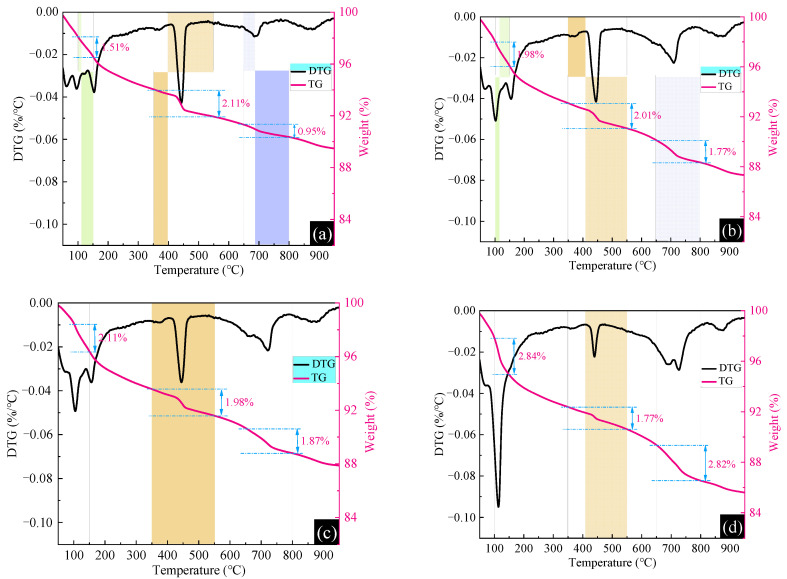
The TG/DTG curves of MTSC. (**a**) MTSC-0, (**b**) MTSC-3, (**c**) MTSC-4, (**d**) MTSC-10.

Figure 9 shows the TG/DTG curves of MTSC-0, MTSC-3, MTSC-4 and MTSC-10. Previous studies showed that the TG/DTG curves of cement hydration specimens are mainly divided into two stages [32,33]. In the first stage, C-S-H, ettringite, AFm and other hydration products lose water and decompose at 30~300 °C. Portlandite (350~550 °C) and carbonate (650~800 °C) decompose in the second stage. The following analysis was carried out by combining the existing studies and the characteristics of MTS. At 100~150 °C, all samples of MTSC primarily undergo decomposition of ettringite [34]. Specifically, the mass losses of ettringite in MTSC-0, MTSC-3, MTSC-4 and MTSC-10 are 1.51, 1.98, 2.11 and 2.84%, respectively. This fully indicates that the gypsum in MTS and cement jointly undergo hydration reactions to produce ettringite. With the increase in MTS content, the ettringite generated in MTSC also increases gradually. At 350~550 °C, all samples mainly experience the decomposition of portlandite [35,36]. It is obvious from Figure 9a–d that the mass loss of portlandite in the samples is in the following order: MTSC-0 > MTSC-3 > MTSC-4 > MTSC-10. The portlandite is mainly generated by the hydrolysis of C_3_S and C_2_S in the cement clinker. However, with the incorporation of MTS, the weak acidity of MTS leads to a sequential decrease in the content of portlandite in MTSC. The sample’s main carbonate decomposition occurs at 650~800 °C [37,38]. It can be seen that the mass loss of carbonate in MTSC gradually increases with the increase in MTS content. This is mainly because, on the one hand, the increase in MTS leads to an excess of gypsum, and part of gypsum does not participate in the reaction. Moreover, the MTS contains other carbonate substances. On the other hand, maybe the irregular operation that occurred during the test process caused the carbonation reaction of MTSC.

**Figure 10:**
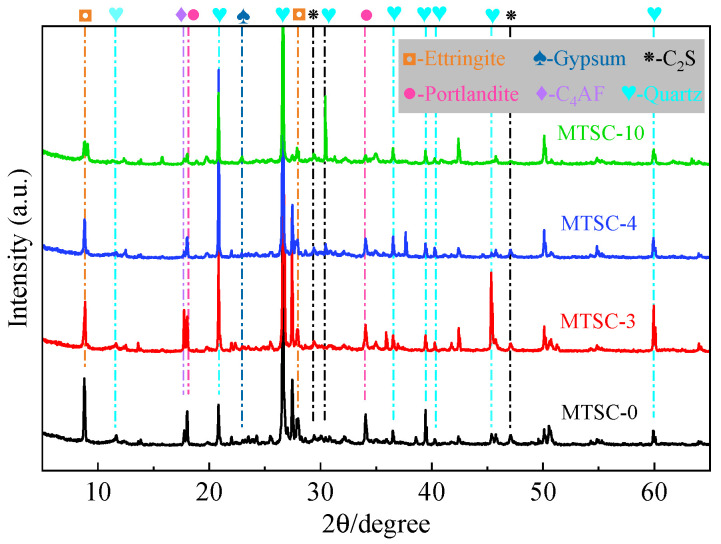
The XRD patterns of MTSC-0, MTSC-3, MTSC-4 and MTSC-10.

The XRD patterns of MTSC-0, MTSC-3, MTSC-4 and MTSC-10 are shown in Figure 10. The main phases of MTSC are quartz, ettringite, portlandite and unreacted cement clinker (C_4_AF and C_2_S). Compared to the concrete without MTS, the incorporation of MTS increases the peak strength of ettringite and decreases the peak strength of portlandite. The results are consistent with the results of TG/DTG curves. It is noteworthy that the gypsum appears in MTSC-10, which indicates that the partial gypsum does not participate in the cement hydration reaction when all RS is replaced by MTS.

In conclusion, the TG/DTG curves and XRD patterns of MTSC show that the increasing dosing of MTS results in the growth of gypsum content, which promotes the formation of ettringite in MTSC. However, the reduced production of portlandite in MTSC is not beneficial to the development of mechanical properties. Therefore, the optimum content of MTS in MTSC should not exceed 40%.

#### 3.4.2. SEM

**Figure 11:**
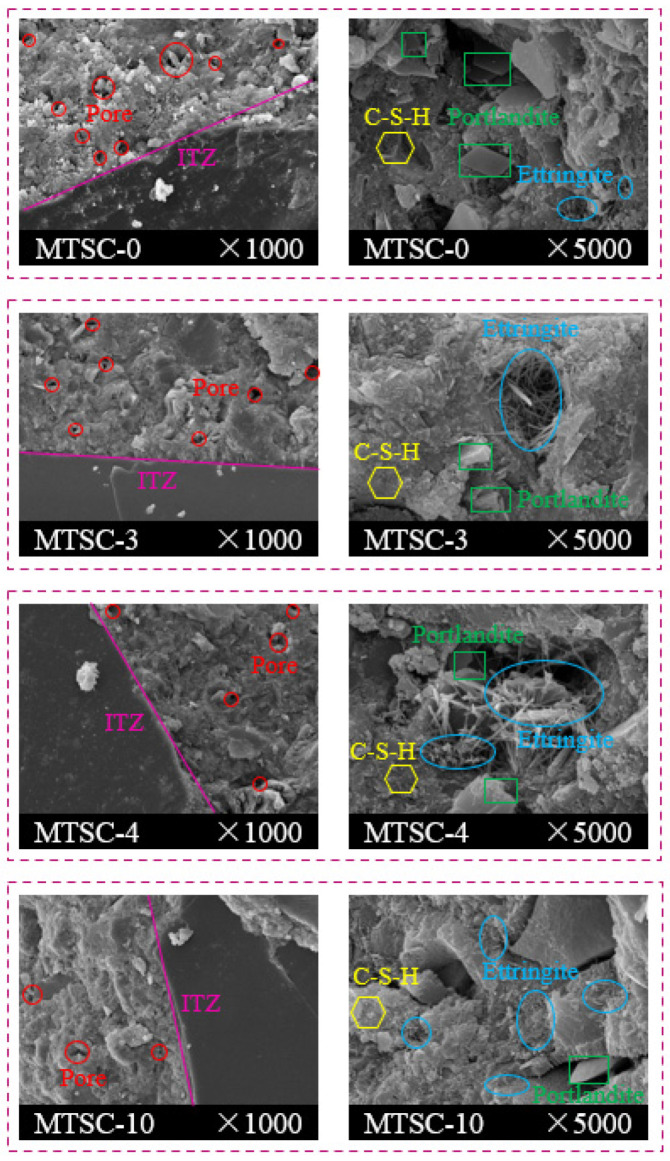
The SEM images of MTSC-0, MTSC-3, MTSC-4 and MTSC-10.

The SEM images of MTSC-0, MTSC-3, MTSC-4 and MTSC-10 are shown in Figure 11. In general, the microstructure of the MTSC is relatively dense. The interfacial transition zones (ITZ), pores, needle-like ettringites, flaked portlandites and C-S-H gels of MTSC can be clearly seen in Figure 11. Specifically, with the increase in MTS content, the SEM images of MTSC show less and less microporosity, which is consistent with the results in Figure 8. MTSC-3 and MTSC-4 produce a large amount of needle-like ettringite compared to MTSC-0. With the increase in needle-like ettringite, the increased ettringite fills the internal voids of the MTSC and makes its structure more compact, which is beneficial to the development of the strength of MTSC. Unlike the needle-like ettringite in MTSC-3 and MTSC-4, the ettringite in MTSC-10 shows a short rod-like uniform distribution. This is mainly due to the interaction between the increased ettringite and the joint extrusion, which causes the morphological transformation of ettringite. Simultaneously, a large amount of ettringite continues to react to generate AFm after cement setting, and it can generate large volume expansion stress, resulting in micro cracks in MTSC microstructure and reducing the mechanical properties of MTSC.

In addition, compared with MTSC-0, the flaked portlandite content in MTSC-3 and MTSC-4 is slightly reduced. The fewer portlandite is generated in MTSC-10. This indicates that the incorporation of excessive MTS reduces the amount of portlandite. This is in full agreement with the results of TG/DTG curves and XRD patterns. Moreover, it was further verified that the preparation of concrete by replacing RS with MTS in an appropriate amount could promote the development of concrete strength. Therefore, the purpose of RS resource saving was achieved.

### 3.5. The Hydration Analysis of MTSC

**Figure 12:**
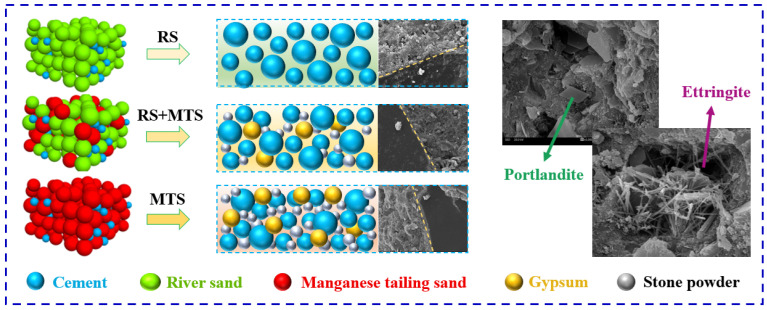
Schematic diagram of concrete prepared with MTS instead of RS.

The substitution of RS by MTS for concrete preparation is an economical and resource-saving method (Figure 12). Therefore, the effect of MTS as solid waste on the hydration of cement deserves to be explored in depth. From the above results, it can be seen that the hydride phase of MTSC does not change significantly, except for the presence of a small amount of gypsum, which is still mainly C-S-H gels, portlandite, ettringite and other incompletely reacted cement clinkers. The effect of MTS dose on cement hydration can be divided into two main parts. The first part is the strengthening phase. A small amount of gypsum phase introduced by MTS (content ≤ 40%) reacts with the C_3_A in the cement [39,40]. The ettringite generated by the reaction fills the pores of MTSC, resulting in a significant reduction in the pores with a diameter less than 0.1 mm and promoting the development of strength in MTSC. The second part is the decay phase. Excessive MTS (content > 40%) introduces a large amount of gypsum and generates too much ettringite. They react with each other and generate expansion stress [41], which causes micro-pores in MTSC to interconnect and form larger pores, making the mechanical properties of MTSC decrease significantly.

The abovementioned studies showed that compared to RS, MTS contains gypsum, which can react with C_3_A in cement by hydration to form ettringite. However, when the content of MTS is low, the ettringite generated by the reaction between gypsum and C_3_A fills the voids of MTSC, which makes its structure dense and enhances the mechanical properties of MTSC, while when the content of manganese tailing sand is high, the reaction generates too much ettringite produces expansion stress, which leads to the strength of MTSC decreasing. Therefore, the preparation of MTSC by replacing RS with an appropriate amount of MTS not only does not reduce the strength of concrete but even improves it. Additionally, the excessive amount of MTS is not suitable to replace RS for preparing concrete.

### 3.6. The Leaching Concentration of Mn^2+^ in MTSC

**Figure 13:**
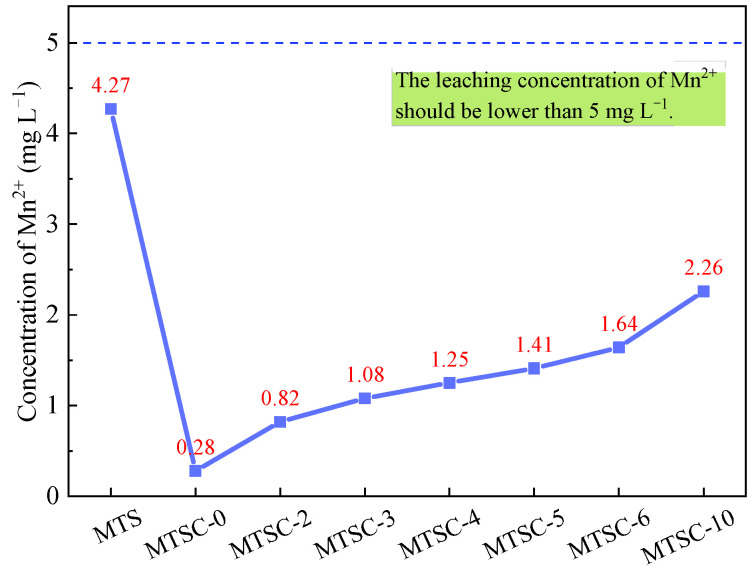
Leaching concentration of Mn^2+^ in MTS and MTSC.

The chemical compositions of MTS (Table 2) indicate that the MnO content in MTS is relatively high. Therefore, there may be a potential risk of manganese leaching in the preparation of concrete from MTS, which may endanger human health and pollute the environment. Studies showed that cement-based materials are capable of solidifying heavy metals [42,43]. Figure 13 displays the leaching concentration of Mn^2+^ in MTS and MTSC. It can be seen that the leaching concentration of Mn^2+^ in MTS is 4.27 mg L^−1^, which meets the requirement of Mn^2+^ concentration of less than 5 mg L^−1^ in the National Standard of the People’s Republic of China: Sewage Discharge Standard (GB/T 8978-1996) [44]. Therefore, the leaching concentration of Mn^2+^ in MTSC prepared by using MTS also meets the requirements. This is consistent with the results in Figure 13. It is worth noting that, compared to MTS, the leaching concentration of Mn^2+^ in MTSC is significantly lower. This is primarily because cement hydration creates an alkaline environment for Mn^2+^ solidification, allowing Mn^2+^ to be converted into stable MnO(OH)_2_ precipitation. Moreover, Mn^2+^ may replace part of Al^3+^ in the C-S-H gel during cement hydration to form a stable structure, thus reducing the leaching of Mn^2+^. In addition, the dense structure of MTSC also prevents the leaching of Mn^2+^, making the leaching concentration of Mn^2+^ in MTSC dramatically lower than that in MTS. Therefore, the use of MTS for concrete preparation can realize the clean treatment of MTS and reduce the risk of environmental pollution from MTS.

## 4. Discussion

It is not feasible to use MTS to replace all RS for concrete preparation. However, when the substitution rate is less than 40%, MTS has little effect on the slump and mechanical properties of concrete and even has a positive effect. There is also no risk of environmental pollution in MTSC.

Moreover, the preparation of concrete with MTS realizes the high-value resource utilization of manganese tailing stone and alleviates the problem of shortage of RS resources. As the MTSC (lower than 40% substitution) exhibited good workability and mechanical properties, it may be recommended for all construction activities. The results are not only in line with the concept of carbon reduction in the concrete industry but also with the current trend in civil and materials engineering.

Although this work provides guidance for the application of MTS in concrete, the effect of MTS on the long-term performance and durability of concrete needs to be further investigated.

## 5. Conclusions

In this paper, the effect of MTS content on the properties and microstructure of MTSC was investigated. The basic properties of MTSC were analyzed by the slump, flexural strength, compressive strength and splitting tensile strength experiments. The pore structure characteristics and microstructure of MTSC were characterized by the hardened concrete pore structure analyzer, TG/DTG, XRD and SEM. The leaching concentration of Mn^2+^ in MTSC was also examined. Based on the results from this study, the following conclusions can be drawn:

(1) When the content of MTS is lower than 40%, the decrease in the concrete slump is insignificant, but when the dosing of MTS exceeds 40%, the slump of concrete decreases obviously;

(2) Compared with MTSC-0, the flexural and compressive strength of MTSC in the early stages (3 d and 7 d) can be improved effectively when the amount of MTS is less than 40%, but the flexural and compressive strength at 28 d not be improved obviously. The flexural and compressive strength of MTSC can be significantly reduced when the amount of MTS is more than 40%. Moreover, with the increase in MTSC content, the splitting tensile strength of MTSC at 28d first remains constant and then decreases;

(3) The incorporation of MTS can reduce the number of pores with a diameter of less than 0.1 mm in concrete and make the pore distribution uniform. However, the excess MTS can cause the number of large pores with a diameter greater than 0.2 mm in concrete to rise sharply. Additionally, the air content in hardened MTSC shows a trend of decreasing and then increasing with the increase in MTS content;

(4) MTS can be hydrated with cement mainly due to the presence of gypsum. With the increase in MTS content, the amount of ettringite in MTSC increases. In addition, excess ettringite interacts with each other to form expansion stress and squeeze the pores, which is not conducive to the development of concrete strength;

(5) The leaching concentration of Mn^2+^ in all MTSC is lower than 5 mg/L. Moreover, cement-based materials can reduce the risk of leaching hazardous substances. Therefore, there is no risk of environmental pollution in the preparation of MTSC by using MTS.

## Figures and Tables

**Table 1:** Physical and mechanical properties of cement.

Specific Surface Area (m^2^·kg^−1^)	Standard Consistency Water (%)	Stability	Setting Time (Min)		Flexural Strength (MPa)		Compressive Strength (MPa)	
			Initial	Final	3 d	28 d	3 d	28 d
4.1	30.2	Qualified	174	224	5.4	6.5	25.1	42.5

**Table 2:** Chemical compositions of cement and MTS.

	SiO_2_	Al_2_O_3_	Fe_2_O_3_	CaO	MgO	SO_3_	MnO	K_2_O	TiO_2_	P_2_O_5_	LOI
Cement	20.14	3.87	3.58	61.47	2.84	2.17	-	-	-	-	3.6
MTS	53.08	16.32	4.68	4.34	1.13	6.02	2.86	3.87	0.65	0.26	5.7

**Table 3:** Physical performance indexes of RS and MTS.

	Stone Powder Content (%)	Clay Lump Content (%)	Apparent Density (g·cm^−3^)	Close Packing Density (g·cm^−3^)	Crushing Index (%)
RS	2.5	1.0	2.63	1.46	17
MTS	24.0	6.9	2.66	1.57	43
Limits	≤10.0	≤2.0	≥2.50	≥1.40	≤30

Note: The limits in Table 3 are based on the provisions of “the national standard of the People’s Republic of China: sand for construction (GB/T14684-2011)” [24].

**Table 4:** Mix proportions of MTSC.

Sample No.	Cement (kg·m^−3^)	RS (kg·m^−3^)	MTS (kg·m^−3^)	CA (kg·m^−3^)	Water (kg·m^−3^)	MTS Content (%)
MTSC-0	350	740	0	1060	175	0
MTSC-2	350	592	148	1060	175	20
MTSC-3	350	518	222	1060	175	30
MTSC-4	350	444	296	1060	175	40
MTSC-5	350	370	370	1060	175	50
MTSC-6	350	296	444	1060	175	60
MTSC-10	350	0	740	1060	175	100

## Data Availability

The data that support the findings of this study are available from the corresponding author upon reasonable request.
